# 231 Wishes of people with excess body weight to physical activity - a qualitative systematic review

**DOI:** 10.1093/eurpub/ckae114.124

**Published:** 2024-09-26

**Authors:** Jeanette Reffstrup Christensen, Rasmus Ostergaard Nielsen

**Affiliations:** Research Unit Of General Practice, Aarhus, Denmark; Research Unit of General Practice, Department of Public Health, University of Southern Denmark, Odense, Denmark; DRIVEN, Institute of Sports Science and Clinical Biomechanics, University of Southern Denmark, Odense, Denmark; Research Unit Of General Practice, Aarhus, Denmark; Department of Public Health, Aarhus University, Aarhus, Denmark

## Abstract

**Purpose:**

The prevalence of people with obesity (Body Mass Index>30) are still rapidly increasing and 1 billion people are now suffering from obesity worldwide. This entails major health challenges for both the individuals and our society, primarily because of increased costs. It is known that physical activity is an effective method to increase health also for people with obesity. But it is also known that people with obesity find it difficult to maintain an increased level of physical activity. The purpose was therefore to identify how physical activity must be planned and organized - also regarding form and content, so it includes the wishes and needs from people with obesity so they can increase and maintain their level of physical activity.

**Materials and methods:**

A qualitative systematic review was registered in the PROSPERO and was conducted according to the Preferred Reporting Items for Systematic Reviews and Meta-Analyses (PRISMA) guidelines. A systematic literature search was carried out in the databases PubMed, CINAHL, MEDLINE, EMBASE, PsykINFO, and Cochrane. The data synthesis was conducted according to Thomas and Harden’s thematic analysis.

**Results:**

The search resulted in 14260 articles after removal of duplicates. Ten articles representing ten different studies were included in the thematic analysis. The articles investigated various phenomena of interest and had been carried out in different countries and cultures. Based on the thematic analysis, two primary themes and seven subthemes emerged.

**Conclusions:**

This qualitative systematic review identified ten articles. The studies covered different aspects and perspectives of people with obesity on how to engage in physical activity. The studies are adding knowledge on how to plan and organize the form and the content of physical activity. These perspectives were synthesised into the two primary themes coping and environment and the seven subthemes health, knowledge and skills, adaptation to everyday life, self-perception and motivation, culture and family support, peers and social aspects, and settings.

**Funding Source:**

No funding source paid for the study except the research units where the two researchers are affiliated.

